# Ferroptosis and the ubiquitin-proteasome system: exploring treatment targets in cancer

**DOI:** 10.3389/fphar.2024.1383203

**Published:** 2024-04-11

**Authors:** Muhammad Azhar Ud Din, Yan Lin, Naijian Wang, Bo Wang, Fei Mao

**Affiliations:** ^1^ Key Laboratory of Medical Science and Laboratory Medicine of Jiangsu Province, School of Medicine Jiangsu University, Zhenjiang, Jiangsu, China; ^2^ Department of Laboratory Medicine, Lianyungang Clinical College, Jiangsu University, Lianyungang, Jiangsu, China; ^3^ The People’s Hospital of Danyang, Affiliated Danyang Hospital of Nantong University, Zhenjiang, Jiangsu, China

**Keywords:** ferroptosis, ubiquitin-proteasome pathway, degradation, cancer, treatment

## Abstract

Ferroptosis is an emerging mode of programmed cell death fueled by iron buildup and lipid peroxidation. Recent evidence points to the function of ferroptosis in the aetiology and development of cancer and other disorders. Consequently, harnessing iron death for disease treatment has diverted the interest of the researchers in the field of basic and clinical research. The ubiquitin-proteasome system (UPS) represents a primary protein degradation pathway in eukaryotes. It involves labelling proteins to be degraded by ubiquitin (Ub), followed by recognition and degradation by the proteasome. Dysfunction of the UPS can contribute to diverse pathological processes, emphasizing the importance of maintaining organismal homeostasis. The regulation of protein stability is a critical component of the intricate molecular mechanism underlying iron death. Moreover, the intricate involvement of the UPS in regulating iron death-related molecules and signaling pathways, providing valuable insights for targeted treatment strategies. Besides, it highlights the potential of ferroptosis as a promising target for cancer therapy, emphasizing the combination between ferroptosis and the UPS. The molecular mechanisms underlying ferroptosis, including key regulators such as glutathione peroxidase 4 (GPX4), cysteine/glutamate transporter (system XC-), and iron metabolism, are thoroughly examined, alongside the role of the UPS in modulating the abundance and activity of crucial proteins for ferroptotic cell death, such as GPX4, and nuclear factor erythroid 2–related factor 2 (NRF2). As a pivotal regulatory system for macromolecular homeostasis, the UPS substantially impacts ferroptosis by directly or indirectly modulating iron death-related molecules or associated signaling pathways. This review explores the involvement of the UPS in regulating iron death-related molecules and signaling pathways, providing valuable insights for the targeted treatment of diseases associated with ferroptosis.

## 1 Introduction

The primary goal of cancer treatment research has been to eliminate malignant cells while protecting healthy cells (Cassetta and Pollard, 2023). To prevent the uncontrolled growth of cancer cells and other abnormal tissues, the body relies on a process known as “regulated cell death” (RCD) to keep things in check (Coradduzza et al., 2023; Huang et al., 2023). Apoptosis, paraptosis, necroptosis, and ferroptosis are among the distinct modes of RCD that have been characterized so far (Tong et al., 2022). The tumor’s development and the treatment response are impacted differently by each RCD subtype. In contrast to necrosis, apoptosis, and paraptosis, ferroptosis is distinguished by ferrous ions’ buildup and lipids’ peroxidation (Su et al., 2019; Zhi et al., 2022; Wu et al., 2023). Ferroptosis has been acknowledged as a promising cellular mechanism with potential anti-tumor benefits since its discovery. Furthermore, it has been connected to several other diseases, such as neurological disorders and harm caused by strokes (Dixon et al., 2012; Gao et al., 2022; Liu et al., 2022). By controlling the metabolism of iron and lipids, ferroptosis regulates the resistance of tumor to chemotherapy or targeted medicines (Qi et al., 2022). The metabolic adaptability of tumor cells, notably their sensitivity to ferroptosis, sheds light on the mechanisms underpinning tumor persistence (Lin et al., 2022). Significant strides have been achieved in increasing cancer cells’ sensitivity to ferroptosis by changing their metabolism or activating oncogenic pathways (Deng et al., 2023). Additionally, it has been found that immunotherapy modifies the efficacy of ferroptosis by increasing the levels of lipid peroxide and iron in tumor cells, which facilitates ferroptosis (Yao et al., 2021).

Among the many molecular mechanisms affecting ferroptosis, the ubiquitin-proteasome pathway is widely involved and is closely related to the occurrence and development of tumors and other diseases (Tang and Kroemer, 2020). The exogenous pathway is initiated by the inhibition of system XC- or activation of transferrin (TF) and lactotransferrin (LTF) on the cell membrane. The endogenous mechanism is triggered by the depletion of intracellular glutathione (GSH) and inactivation of GSH peroxidase 4 (GPX4) (Ingold et al., 2018). Ferroptosis plays a crucial role in the development and diseases (Distéfano et al., 2017; Jiang et al., 2021), and has become a hot spot in biological research in recent years. Therefore, understanding the relationship between the UPS and ferroptosis can help understand the complex mechanism of ferroptosis and provide new strategies for treating human diseases, including tumors (Chen et al., 2021), neurodegenerative diseases (Masaldan et al., 2019), cardiomyopathy (Fang et al., 2019) ischemia-reperfusion injury (Li et al., 2020), stroke (Alim et al., 2019), traumatic brain injury of cerebral hemorrhage (Bao et al., 2021), ageing (Timmers et al., 2020), atherosclerosis (Ouyang et al., 2021), liver injury (Chen et al., 2022), and kidney injury (Tonnus et al., 2021), or related pathological cell death. With the development of molecular biology, various regulatory factors and related mechanisms mediating ferroptosis are being revealed, and the basic research of ferroptosis is gradually deepening. The ubiquitin-proteasome pathway plays a significant role in ferroptosis and has been linked to the development of cancer and other diseases. Therefore, comprehending the connection between the UPS and ferroptosis will aid in understanding the intricate process of ferroptosis and offer fresh approaches for treating human disorders, such as tumor.

## 2 Distinctive features of ferroptosis

**FIGURE 1:**
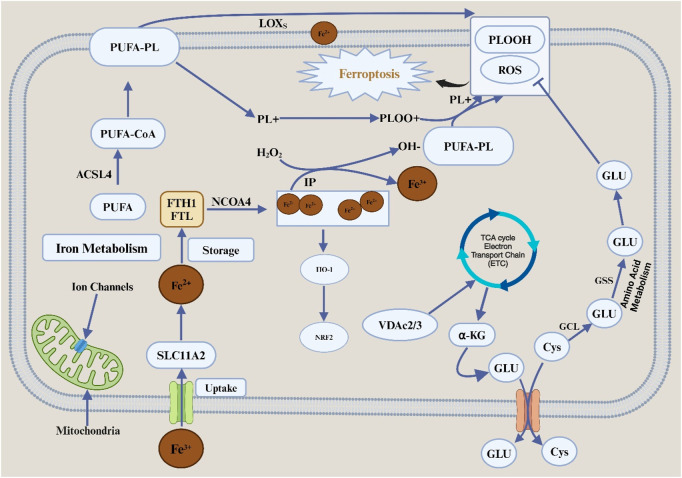
Features and mechanisms of ferroptosis. Abbreviation: TFRC, transferrin receptor; SLC11A, solute carrier family 11 member 2; FTL, ferritin light chain; FTH1, ferratin heavy chain 1; NCOA4, nuclear receptor coactivator 4; HO-1, heme oxygenase 1; NRF2, nuclear factor erythroid2-related factor 2; IP, iron pool; PUFA, polyunsaturated fatty acid; CoA, coenzyme A; PL, phospholipids; LOXs, lipid oxygenase; PLOOH, phospholipid hydroperoxides; Cys, cystine; Glu, glutamate; SLC7A11, solute carrier family 7 member 11; SLC3A2, solute carrier family 3 member 2; GCL, glutamate-cystine ligase; GSS, glutathione synthetase; GSH, glutathione; GPX4, glutathione peroxidase 4; ROS, reactive oxygen species; TCA, tricarboxylic acid; VDAC2/3, voltage-dependent anion channel 2/3; α-KG, alpha ketoglutaric acid; ETC, electron transfer chain.

Ferroptosis is defined by mitochondrial dysfunction and cell death due to iron-dependent lipid peroxidation. It is characterized by several morphological changes, including the blistering of the plasma membrane, the reduction or absence of the mitochondrial ridges, and the preservation of the usual size of the nucleus without chromatin condensation (Rodriguez et al., 2022). The delicate lipid peroxide balance within the cell plays a critical role in the incidence of ferroptosis. Metabolic pathways tightly control ferroptosis, including lipids, iron, and amino acids Figure 1.

Ferroptosis is induced by the iron-mediated oxidation of polyunsaturated fatty acid (PUFA). Regarding biological properties, ferroptosis cell death is associated with the formation of reactive oxygen species (ROS), evidenced mainly by iron and lipid peroxidation buildup. When the metabolism of the antioxidant system in cells is disturbed, it causes an increase in the accumulation of Fe^2+^ within the cell, which in turn mediate the Fenton reaction to produce hydroxyl (-OH) or alkoxy (R-O) free radicals with different peroxides (Su et al., 2019) which can peroxidize with unsaturated fatty acids on the cell membrane. It causes the destruction of the stability of the lipid bilayer and the disintegration of the cell membrane, thus promoting ferroptosis in cells. The mitochondrial morphology of cells undergoing ferroptosis changes significantly, including mitochondrial volume reduction, membrane density increases and ridge reduction or even disappearance. Compared with apoptosis, the nuclear structure of cells undergoing ferroptosis is complete, without nuclear membrane rupture and chromatin marginalization. It is also accompanied by cell shedding and aggregation (Li and Li, 2020).

The occurrence or inhibition of ferroptosis is associated with various molecular mechanisms closely related to the metabolism of amino acids, lipids, iron and other metabolic modes in the cell. The regulatory mechanisms of ferroptosis can be divided into positive and negative ones. Iron metabolism is the essential positive regulatory mechanism of ferroptosis. As mentioned above, excessive accumulation of iron ions in cells can promote the occurrence of ferroptosis. Extracellular Fe^3+^ is endocytosed into the cell body by transferrin receptor protein 1 (TFR1). After the reduction of Fe^3+^ into ferrous ion Fe^2+^ in the cell, some Fe^2+^ is further stored in the unstable iron pool mediated by DMT1 (divalent metal transporter 1) or zinc-regulated, iron-regulated transporter-like proteins 8 ZIP8/14 (ZRT/IRT-like proteins 8/14). The other part is stored in an iron storage protein complex composed of FTL (ferritin light chain) and FTH1 (ferritin heavy chain). Excessive accumulation of Fe^2+^ triggers the Fenton reaction, which promotes cell ferroptosis.

The SLC7A11-GSH-GPX4 pathway is considered to be the primary negative regulatory mechanism in cells (Lei et al., 2022). In mammal cells, SLC7A11, a member of system XC- on the cell membrane, can take cystine into the cell to synthesize GSH, which is a necessary component for the activity of GPX4, a vital lipid peroxidase in the cell, so that GPX4 can directly reduce phospholipid hydrogen peroxide to hydroxy phospholipid. It prevents the peroxidation of PUFA on the cell membrane and thus inhibits ferroptosis in cells (Koppula et al., 2021). At the same time, more studies have shown that there are non-GPX4-dependent harmful regulatory mechanisms of ferroptosis in cells, such as the ferroptosis suppressor protein (FSP1-CoQH2 system), human dihydroorotate dehydrogenase (DHODH-CoQH2 system) and GCH1-BH4 system. Among them, FSP1, as a nicotinamide adenine dinucleotide phosphate [NAD(P) H-dependent oxidoreductase], can reduce CoQ (CoQ) to panthenol (CoQH2), thereby trapping lipid peroxy radicals and inhibiting lipid peroxidation and ferroptosis (Bersuker et al., 2019; Doll et al., 2019). Additionally, one study discovered the first ferroptosis defense mechanism operating within mitochondria: dihydroorotate dehydrogenase (DHODH), an enzyme involved in pyrimidine synthesis, converts CoQ to CoQH2 in the inner mitochondrial membrane, neutralizing the lipid peroxidation defense against ferroptosis in mitochondria (Mao et al., 2021). Through the synthesis of BH4 (tetrahydrobiopterin), a free radical-scavenging antioxidant, CoQH2, and phospholipids (FLs) with two PUFA tails, GCH1 (GTP cyclohydrolase 1) prevents ferroptosis in the GCH1-BH4 system (Soula et al., 2020). Additionally, several transcription factors involved in ferroptosis, including tumor protein 53 (TP53), NFE2L2/NRF2, and activating transcription factor (ATF3), have diverse functions in modulating ferroptosis sensitivity using transcription-dependent or transcription-independent techniques (Dai et al., 2020).

### 2.1 Ferroptosis and iron metabolism

Ferroptosis, the process of acquiring, using, storing, and eventually excreting iron, relies heavily on iron metabolism (Hu et al., 2021). During digestion, the duodenum’s epithelial cells absorb iron from meals. DMT1 then transports Fe^2+^ into the cells after iron reductase converts Fe^3+^ in the intestinal epithelium (Richardson and Ponka, 1997). Iron homeostasis in the body is crucial for maintaining the balance of this essential element. Transferrin and ferritin play vital roles in this process. Transferrin is a glycoprotein that binds to Fe^3+^ in the blood, forming a complex. This complex is taken up by cells through endocytosis mediated by the TfR (Anderson and Frazer, 2017; Yanatori and Kishi, 2019; Shamsi et al., 2020; Qiu et al., 2022). Once inside the cell, the iron is released from transferrin in the acidic environment of the endosome. This release is facilitated by a combination of low pH, a conformational change in transferrin upon binding to its receptor, and reduction of transferrin-bound Fe^3+^. The released iron can then be used for metabolic functions, stored within cytosolic ferritin, or exported from the cell via ferroportin (FPN1) (Anderson and Frazer, 2017). Ferritin, an intracellular protein, serves as the primary storage site for excess iron. It accumulates and sequesters iron, preventing its potential neurotoxic effects. Ferritin molecules can accumulate excess iron and are engulfed by lysosomes in a process called “autophagy” (Arosio et al., 2017). Together, transferrin and ferritin play critical roles in maintaining iron homeostasis. Transferrin transports iron to cells, while ferritin stores excess iron, preventing its toxicity. This intricate interplay ensures the proper distribution and storage of iron, supporting various physiological processes in the body (Anderson and Frazer, 2017). An iron pool (IP) that is non-toxic to cells can be formed when iron is safely stored in ferritin. Compared to the IP, the cell’s free Fe^2+^ concentration is substantially lower (Lin et al., 2020; Philpott et al., 2020). Free Fe^2+^ is highly reactive and readily forms hydroxyl free radicals when combined with intracellular hydrogen peroxide (H_2_O_2_). This causes oxidative damage to DNA, proteins, and membrane lipids. This pathway promotes lipid oxidation, which harms cell membranes and eventually results in cell death (Hong et al., 2021; Qin et al., 2023). Additionally, by lowering the amount of redox-active iron available, several mitochondrial proteins, such as NFS1-ISCU26, CISD1, and CISD2, can negatively control ferroptosis (Chen et al., 2021). There are two parts of ferritin, the FTH1 and the FTL (Zhang et al., 2021). Ferritinophagy is the process whereby ferritin degrades and releases Fe^2+^ (Sun et al., 2022). To create a ferritin complex, the nuclear receptor coactivator 4 (NCOA4) can attach to FTH1 directly. An increase in intracellular free Fe^2+^ hastens ferroptotic cell death, an increase in the concentration of ferritin phagosomes, and an increase in NCOA4 (Jin et al., 2022; Mi et al., 2023).

Another typical mechanism for ferritin breakdown in cardiomyocytes is heme catalysis via heme oxygenase-1 (HO-1). NRF2 and BAY have been suggested to activate this pathway (Wang et al., 2022; Xu et al., 2023). Researchers have found that increasing HO-1 expression accelerates elastin-induced ferroptosis (Guan et al., 2022). Most cells do not have an efficient method to remove iron when it accumulates beyond storage capacity, which results in elevated levels of the labile IP. A higher concentration of free iron within the cell stimulates the production of labile IP, which speeds up the onset of ferroptosis (Ou et al., 2022).

### 2.2 Role of lipid metabolism in ferroptosis

PUFAs are essential to the fluidity of cell membranes because of their location in the phospholipid bilayer. An overabundance of PUFAs can cause the Fenton reaction to convert them into hydroxyl radicals, which in turn can cause an overabundance of lipid peroxide and, in turn, cause cells to enter ferroptosis (Dierge et al., 2021; Mishima and Conrad, 2022). Phosphatidyl ethanolamine (PE) makes up roughly 15%–25% of the phospholipids in the membranes of other organelles compared to about 40%–45% of the total phospholipids in the inner membrane of mitochondria (Feng and Stockwell, 2018; Yi et al., 2021). Data indicates that PE contributes to the ferroptosis triggered by arachidonic acid (AA) and its analogues. Acyl-CoA synthetase long-chain family member 4 (ACSL4) catalyzes the reaction in which PUFAs attach to coenzyme A (CoA) to form Acyl-CoA, a key intermediate in the non-enzymatic process of lipid peroxidation (Bouchaoui et al., 2023). Acyl-CoA then undergoes re-esterification to create phospholipids, a process performed by lysophosphatidylcholine acyltransferase 3 (LPCAT3) (Xu et al., 2022). The membrane remodeling enzymes ACSL4 and LPCAT3 are crucial for promoting ferroptosis (Feng et al., 2022). They control the levels of PUFAs in phospholipids.

PE and LPCAT3 boost ferroptosis by inducing membrane phospholipid peroxidation via lipid oxygenase (LOX) activity. Therefore, protecting cells against elastin-induced ferroptosis can be achieved by silencing LOXs (Lee et al., 2022). Multiple mechanisms exist by which lipid peroxides cause damage to cells, including the generation of ROS through the amplification of lipid peroxidation processes, alterations to the membrane’s physical structure, and lipid peroxidation byproducts (Zhan et al., 2022).

### 2.3 Connection of ferroptosis to amino acid metabolism

One of the most effective antioxidants is GSH (Yang et al., 2022). It helps repair damaged cell membranes by acting as a GPX4 substrate, part of the body’s natural defense mechanism. An essential transporter for GSH production is the cystine/glutamate antiporter (XC-) (Wu et al., 2021; Du and Guo, 2022). The system XC- and GPX4 are both essential for the amino acid metabolism associated with ferroptosis. There is only one cellular component, selenocysteine, that can convert lipid peroxide to the equivalent alcohol, and it is located in the center of GPX4. Ferroptosis can occur when GPX4 cannot remove hydrogen peroxide from the environment (Luo et al., 2022; Ma et al., 2022). The system XC- allows cystine and glutamate to be imported and expelled from cells, respectively. To aid in the production of GSH, cystine is converted to cysteine by the internal enzymes glutamate-cysteine ligase (GCL) and glutathione synthase (GSS). Heterodimers of the solute carrier family 3 member 2 (SLC3A2) and the system XC- are responsible for transporting cystine and glutamic acid, respectively (Li et al., 2022). Inducing ferroptosis is possible either by blocking the system XC- directly or indirectly via elevated extracellular glutamate levels.

Additionally, recent research suggests that tryptophan metabolites play a role in the resistance of tumor cells to ferroptosis, a type of cell death distinct from cysteine-mediated ferroptosis. These metabolites include 3-hydroxycyananilic acid (3-HA) and 5-hydroxytryptamine (5-HT). As a result, blocking tryptophan metabolism may be an effective new strategy in the fight against cancer (Liu et al., 2023).

## 3 The ubiquitin-proteasome system (UPS) pathway

**FIGURE 2:**
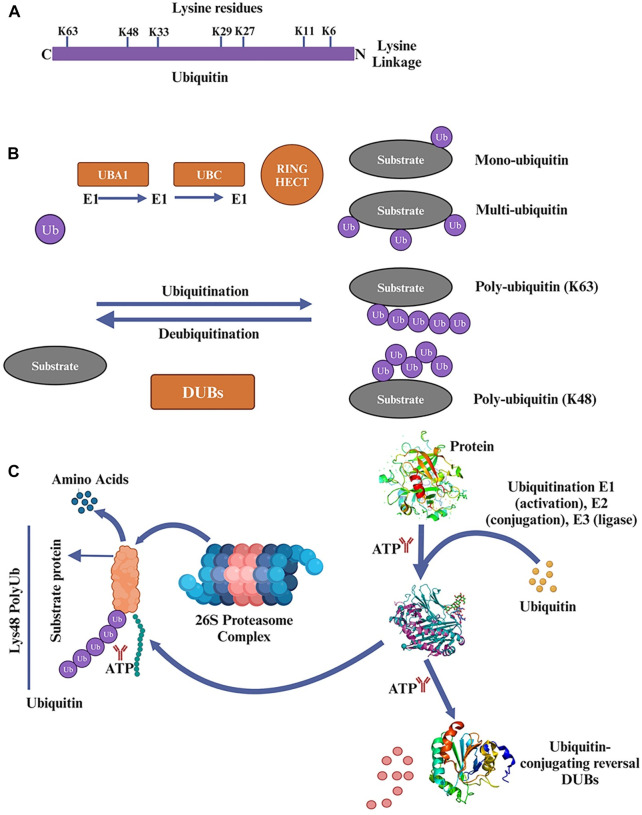
**(A)** Ubiquitin 76 amino acids that is found in various organisms and is highly conserved throughout evolution. It plays a crucial role in ubiquitination, which is involved in regulating protein degradation, cellular signaling, and other important cellular functions. Ubiquitin contains lysine residues (K6, K11, K27, K29, K33, K48, and K63) that are crucial for forming different types of ubiquitin chains and determining the fate of the target protein. **(B)** In the ubiquitination process, ubiquitin is attached to lysine residues of substrate proteins through a three-step enzymatic reaction. First, ubiquitin is activated by a protein called ubiquitin-activating enzyme (E1). Then, the activated ubiquitin is transferred to another protein called ubiquitin-conjugating enzyme (E2). Finally, a protein called ubiquitin ligase (E3) transfers the ubiquitin from E2 to a lysine residue on the target protein. Mono- and polyubiquitin serve as signals in cellular processes such as endocytosis, DNA repair, apoptosis, and transcriptional regulation. Ubiquitin conjugation plays a crucial role in multiple pathways and is not limited to protein degradation alone. **(C)** The ubiquitin-proteasome system (UPS) is a process in which substrate proteins are ubiquitinated through the action of three enzymes: E1, E2, and E3. E1 binds to activated ubiquitin and transfers it to E2, which carries the ubiquitin to E3. E3 then facilitates the transfer of ubiquitin from E2 to a lysine residue on the target protein. Proteins can undergo modification with a single mono-ubiquitin molecule or with ubiquitin chains of varying lengths and linkage types. Substrate proteins modified with specific chains are recognized and degraded by the 26S proteasome. Deubiquitinating enzymes (DUBs) play a role in removing ubiquitin from substrate proteins. DUBs can remove mono-ubiquitination or trim/remove ubiquitin chains. Poly-ubiquitination is typically associated with protein clearance through proteasomal degradation, while mono-ubiquitination affects cellular processes by adding a single ubiquitin moiety to the substrate protein.

Eukaryotic cells rely heavily on the UPS to regulate various cellular processes, including protein homeostasis, cell cycle progression, and response to stress, signal transmission, and transcriptional activation (Myung et al., 2001; Lan et al., 2018). In eukaryotic cells, intracellular proteins that are oxidized, damaged, or misfolded are broken down by UPS in around 80% of cases (Yang et al., 2021). Although UPS and autophagy are essential mechanisms for protein degradation, the size of the degraded materials strongly influences the degradation pathway employed (Dikic, 2017). The UPS typically breaks down unfolded polypeptides, whereas more enormous cytosolic complexes, cellular aggregates, and organelles are handled by autophagy. A group of small peptides with 76 amino acids known as ubiquitin molecules contains lysine residues (K6, K11, K27, K29, K33, K48, and K63) Figure 2A, which are activated by the enzymes E1 (ubiquitin-activating enzyme), E2 (ubiquitin-conjugating enzyme), and E3 (ubiquitin-protein ligase), are used in covalent bonding to the target protein for alterations during the catalytic cascade (Glickman and Ciechanover, 2002). Monoubiquitination and polyubiquitination are the two basic types of protein ubiquitination. A single ubiquitin molecule is covalently joined to a lysine residue in the target protein during monoubiquitination, the most basic type of ubiquitination (Tonnus et al., 2021). K6, K11, K33, K29, K27, K48, and K63 are polyubiquitination alterations. K48 and K63 changes have received the most research attention of them Figure 2B. The 26S proteasome, which controls protein stability, can identify and eliminate the polyubiquitination of K48.

In contrast, the role of K63 is mainly involved in DNA repair, mediating signal transduction and regulating protein activity (Walczak et al., 2012; Yau and Rape, 2016). In addition to the above ubiquitin modifications, there is also a type of ubiquitin chain used to modify proteins, the linear ubiquitin chain (M1 type), which is covalently linked to the N-terminal methionine residue of one ubiquitin and the C-terminal glycine residue of another. Linear ubiquitin chain synthesis and degradation are distinct processes (Shabek and Ciechanover, 2010). Only the E3 ubiquitin ligase HOIP, along with its regulatory subunits HOIL1L and SHARPIN, can facilitate the synthesis of linear ubiquitin chains, hence the name “linear ubiquitin chain assembly complex” (LUBAC). Deubiquitinating enzymes (DUBs) such as OTULIN (OTU deubiquitinase with linear linkage specificity) remove ubiquitin chains from their targets (Jahan et al., 2021).

The covalent binding of ubiquitin, a tiny protein with 76 amino acids, to the protein substrate, is a crucial step in the complex and well-coordinated process of targeted protein destruction (Nandi et al., 2006). Three enzymes work together in sequence to accomplish this. The E1 requires the energy released by the breakdown of ATP to activate itself, which then causes the ubiquitin molecule to establish a thioester bond with E1. Once again, creating a thioester bond like the first, ubiquitin is transported from E1 to the E2. E3 finally catalyzes the covalent attachment of ubiquitin to the target protein’s lysine residues (Fang and Weissman, 2004).

This process is greatly aided by the 26S proteasome complex, which consists of a core 20S proteasome and one or two regulatory 19S proteasome units. When a target protein is modified with a polyubiquitin chain, the 19S proteasome recognizes it, digests the polyubiquitin chain, unfolds the protein, and then translocates it to the 20S proteasome where it is broken down into short peptides (Adams, 2003). In contrast to polyubiquitination, which often results in the proteasomal destruction of proteins, monoubiquitination, in which a single ubiquitin unit is added to the substrate protein, has been shown to affect several cellular functions (Miranda and Sorkin, 2007; Wang et al., 2012). Some examples are protein translocation, DNA damage signaling, epigenetic regulation, and kinase activity as shown in Figure 2C.

Protein ubiquitination is often reversible, and proteins modified by ubiquitination can also be removed by DUBs (Lange et al., 2022). Current studies have shown that the human genome can encode more than 100 deubiquitination enzymes, which are divided into two categories: cysteine proteinases and metalloproteinases. Cysteine proteases can be subdivided into USPs (ubiquitin-specific proteases), MJDs (machadoJoseph disease domain superfamily), OTUs (otubain/ovarian tumor-domain containing proteins), UCHs (ubiquitin carboxyl-terminal hydrolases), MINDYs (motif interacting with each other) Ub-containing novel DUB family), ZUP1 (zinc finger-containing ubiquitin peptidase 1) has six families (Clague et al., 2019). In addition to reversing the ubiquitin modification of the substrate protein, DUBs also participate in the editing of the ubiquitin chain, the recovery of ubiquitin molecules, and the processing and maturation of ubiquitin precursors (Haq and Ramakrishna, 2017). Therefore, the abnormal expression or activation of deubiquitination enzymes in cells will directly or indirectly disrupt the regulation of the corresponding signaling pathways, leading to various diseases such as tumors.

The UPS is made up of the 26S proteasome, ubiquitin, enzymes that activate, bind, link, deubiquitinate, and deubiquitinate ubiquitin, as well proteins broken down by 26S proteasome. Research has shown that the UPS is crucial for protein degradation in cells and that it can destroy up to 80%–85% of proteins in eukaryotic cells (Amm et al., 2014). When a ubiquitin tag is added to a substrate, it can alter the substrate’s function, localization, protein interaction, or stability and then regulate many different cellular life activities (Lu et al., 2013; Streich Jr and Lima, 2014). These activities are crucial to cell growth. Diseases including cancer, Alzheimer’s, Parkinson’s, Huntington’s, amyotrophic lateral sclerosis (ALS), spongiform encephalopathy (SLE), and arrhythmia can all be effectively treated by inhibiting the proteasome system (Pohl and Dikic, 2019).

## 4 The UPS and ferroptosis

### 4.1 The UPS and iron transport system

Factors related to iron metabolism play an essential role in ferroptosis and are potential targets to induce ferroptosis in cells. Studies have shown that in colon cancer, OTUD1 can bind and promote the deubiquitination modification of IREB2 (iron-responsive element-binding protein2), a primary regulator of iron metabolism. Promote the stability of IREB2 protein and activate the expression of its downstream TFRC gene, resulting in intracellular Fe^2+^ aggregation and increased ROS level, and eventually raise colon cancer cells’ susceptibility to ferroptosis. It also encourages the release of DAMPs (DAM-associated molecular patterns) to draw in white blood cells and strengthen the host’s immune response (Song et al., 2021). Lactoferrin (LF) is a glycoprotein transferrin with the unique ability to bind and transport iron. LTF, like transferrin, increases iron uptake, encouraging ferroptosis in ovarian and pancreatic cancer cells. When RSL3 and Erastin activate ferroptosis *in vitro*, iron buildup and oxidative damage are decreased because the E3 ubiquitin ligase NEDD4L mediates the protein degradation of LTF (Wang et al., 2020). Ferroportin is an essential transferrin for the balance of intracellular iron metabolism, responsible for the outward transport of iron, and it is also the only iron transporter found in mammals (Ganz, 2005; Kuang and Wang, 2019). Studies have shown that USP35 is significantly overexpressed in human lung cancer cells and tissues, and USP35 can maintain the stability of FPN protein by targeting FPN. Functionally, knocking down USP35 in lung cancer cells can directly promote ferroptosis by down-regulating FPN protein levels and inhibiting cell growth and clone formation. In addition, under ferroptosis activator Erastin or RSL3 stimulation, knocking down USP35 can cause iron metabolism disorder and pig ferroptosis of lung cancer cells, thus promoting the growth of lung cancer cells and tumor progression (Tang et al., 2021).

### 4.2 The UPS and SLC7A11

Studies have shown that the deubiquitinating enzyme OTUB1, which can bind the CD44 (cluster of differentiation-44), is recruited under the effect of removing the ubiquitin chain on SLC7A11 to prevent its degradation through the proteasome and improve its stability, thereby inhibiting the occurrence of ferroptosis in tumors (Liu et al., 2019). However, the essential E3 ligase that causes SLC7A11 degradation in human tumors is unknown. SLC7A11 is regulated by TP53, NFE2L2/NRF2, ATF3, ATF4, BACH1 and other transcription factors and epigenetic regulation. Therefore, in addition to being directly regulated by the proteasome pathway at the protein level, SLC7A11 is also indirectly affected by the proteasome pathway through transcription factors and regulates ferroptosis. Studies have shown that in glioma cells, activation of the KEAP1-NRF2 signaling axis upregulates SLC7A11 and promotes glutamate secretion, thereby affecting the tumor microenvironment and promoting cell proliferation and resistance to ferroptosis (Fan et al., 2017). In lung cancer cells, p53 can promote the nuclear translocation of the deubiquitinating enzyme USP7, which further removes the ubiquitination of histone H2B from the SLC7A11 gene’s regulatory area, thus inhibiting the transcription of SLC7A11 and inactivating the expression of SCL7A11, thereby encouraging ferroptosis in cells (Wang et al., 2019). In addition, the deubiquitinating enzyme BAP1 can remove monoubiquitination at the lysine 119 site of histone H2A on the SLC7A11 promoter, thereby inhibiting SLC7A11 transcription, further reducing cystine uptake of cells and increasing ferroptosis sensitivity. This process is independent of p53 (Wang et al., 2004; Zhang et al., 2018).

Further studies have shown that PRC1 (Polycomb Repressive Complex 1) can ubiquitinate histone H2A using Ring1B, Bmi1, and an E2 ubiquitin-binding enzyme UbcH5c. The SLC7A11 promoter’s H2A is ubiquitinated, which inhibits SLC7A11’s transcription. BAP1 and PCR1 can work together to simultaneously control the promoter’s level of ubiquitination, which in turn controls SLC7A11 production (Zhang et al., 2019a). It is not ruled out that BAP1 and PRC1 may be involved in regulating ferroptosis through synergistic regulation of SLC7A11.

### 4.3 The UPS and GPX4

Studies have shown that PdPT, a broad-spectrum inhibitor of deubiquitination enzyme, induces ferroptosis by promoting the degradation of GPX4 protein in non-small cell lung cancer cells and further inhibits tumor growth (Yang et al., 2020). The specific mechanism is that PdPT can inhibit the deubiquitination of GPX4 by a variety of deubiquitination enzymes, including USP family members (USP14, USP15, USP10, USP7, and USP25) and UCH family members UCHL5, resulting in ubiquitination degradation of GPX4. However, the proteasome inhibitor bortezomide can reverse PDPT-induced GPX4 degradation, suggesting that the proteasome plays an essential role in the ferroptosis induced by PDPT-induced GPX4 degradation (Dong et al., 2022). In addition, by analyzing the changes in different types of ubiquitination modifications in cell ferroptosis, it has been found that cell ferroptosis promotes the increase of intracellular linear ubiquitination levels, and linear ubiquitination can regulate cell ferroptosis. When stimulated by the ferroptosis activator RSL3, GPX4 is ubiquitinated in large quantities, thus recruiting a more LUBAC. LUBAC enhances the protein stability of GPX4 by regulating its linear ubiquitination level and delaying the occurrence of cell ferroptosis, thereby inhibiting cell ferroptosis. The *in vitro* deubiquitination reaction system was used to analyze the ubiquitination status of GPX4 during ferroptosis. It was found that there were various types of ubiquitination modifications in GPX4, among which K63 ubiquitination was the primary type, and M1 and K48 ubiquitin chains could be linked to K63 ubiquitin chains to form hybrid ubiquitin chains. OTUD5 can remove the K63 ubiquitin chain of GPX4, reduce LUBAC recruitment, decrease linear ubiquitination level, destroy the protein stability of GPX4, and further affect ferroptosis of cells.

### 4.4 The UPS and NRF2

A transcription factor called NRF2 is essential for regulating antioxidant genes and managing cellular REDOX equilibrium. Under normal physiological conditions, cells undergo a process where NRF2 is kept in a liquid state. This occurs when NRF2 binds to its regulatory molecule known as KEAP1 (Kelch-like ECH-associated protein 1) within the cytoplasm. Following this binding, NRF2 is transported to the proteasome, a cellular structure responsible for protein degradation. However, when the cells are subjected to oxidative stress and electrophilic stimulation, NRF2 can be activated. The cysteine residues on the structure of KEAP1 will bind to intracellular ROS or electrophiles, causing conformational changes, during which NRF2 dissociates from KEAP1. Stable NRF2 undergoes nuclear translocation and interacts with antioxidant response elements (ARE) in the target gene promoter region to activate the expression of many cell protection genes, affecting various cell biological functions, such as apoptosis and senescence (Yamamoto et al., 2018). The role of the KEAP1-NRF2 signaling pathway in maintaining appropriate REDOX homeostasis has been well established, and there is growing proof that KEAP1-NRF2 is closely related to the regulation of ferroptosis. For example, recently, researchers using CRISPR screening found that KEAP1 is a key regulator of ferroptosis and showed that the lack of KEAP1 can enhance the resistance of cells to ferroptosis (Cao et al., 2019). This mechanism may be through the p62-KEAP1-NRF2 pathway to upregulate the expression of multip

le genes involved in iron and ROS metabolism (such as SLC7A11, NQO1, HO1 and FTH1), thereby inhibiting cell ferroptosis (Sun et al., 2016b). When NRF2 is strictly regulated by the E3 enzyme KEAP1, it has a corresponding cell stabilization mechanism. Studies have shown that by maintaining the stability of the NRF2 protein, the deubiquitination enzyme USP11 controls ferroptosis and cell division in non-small cell lung cancer (Meng et al., 2021). With the increasing research on applying the ferroptosis mechanism in tumors, NRF2 is also an essential target for tumor treatment. Studies have shown that blocking NRF2 enhances the *in vitro* and *in vivo* anti-cancer activity of ferroptosis inducers, including Erastin and sorafenib (Meng et al., 2021). A further benefit of NRF2 activation is stopping ferroptosis from damaging renal tissue (Hu et al., 2022). In addition to inducing ferroptosis *in vitro* when stimulated by RSL3, Erastin, and ML162 (Dodson et al., 2019), NRF2 also suppresses the expression of genes that regulate GSH release, such as ABCC1/MRP1. These results suggest that most of NRF2’s actions in ferroptosis are transcription-dependent; through its transcription factors, it controls the transcription of genes involved in ferroptosis before impacting ferroptosis. However, investigations have yet to be done on its transcription-independent roles in ferroptosis. NRF2 target genes can regulate cells’ antioxidant, iron, and intermediate metabolic states in the context of disease therapy.

Additionally, it has been established that NRF2 regulates GPX4 and SLC7A11, the two critical targets for suppressing ferroptosis (Shin et al., 2018). These two molecules also serve to regulate ferroptosis further. Therefore, it is still possible to target NRF2 in disorders marked mainly by ferroptosis and lipid peroxidation.

### 4.5 The UPS and KEAP1-NFE2L2

In ferroptosis, the NFE2L2/NRF2 system functions as a transcriptional defense system (Dai et al., 2020). Because it binds to Kelch-like ECH-associated protein 1 (KEAP1), the transcription factor NFE2L2 is constitutively degraded by the UPS during quiescent conditions. When electrophilic stress and oxidative stress occur, KEAP1 is inactivated, stabilizing NFE2L2 and triggering the transcriptional activation of several cytoprotective genes. The autophagy receptor Sequestosome 1 (SQSTM1/p62) can interact with KEAP1’s NFE2L2-binding site to stabilize NFE2L2 (Komatsu et al., 2010). Eratin and sorafenib, two ferroptosis activators, cause an increase in the interaction between SQSTM1 and KEAP1, which in turn causes an accumulation of NFE2L2 and enhanced nuclear transcription of NFE2L2 target genes, including FTH1, SLC7A11, NAD(P)H quinone dehydrogenase 1 (NQO1), and heme oxygenase 1 (HMOX1) (Sun et al., 2016b; Anandhan et al., 2020). Blocking NFE2L2 consistently increases the anticancer activity of ferroptosis inducers *in vitro* and *in vivo*, including sorafenib, a first-line medication for patients with liver cancer (Sun et al., 2016a; Sun et al., 2016b). NFE2L2 activation, on the other hand, might stop ferroptotic tissue damage. Moreover, NFE2L2 suppresses GSH release-related genes [such as ATP-binding cassette sub family C member 1 (ABCC1/MRP1)] to enhance ferroptosis *in vitro* brought on by RSL3, erastin, and ML162 (Cao et al., 2019). While nothing is known about NFE2L2’s transcription-independent role in ferroptosis, all of these studies point to a transcription-dependent role for the protein in the process.

## 5 The promise of directing therapies towards ubiquitin enzymes and ferroptosis

Ferroptosis-based treatments have produced intriguing results in cancer experimentation. Increasing amounts of evidence point to the possibility that cancer cells’ production of ferroptosis could represent a brand-new avenue for clinical intervention (Wang et al., 2021). Unfortunately, the cancer patient community has not yet fully realized the potential of medicines producing ferroptosis because of a lack of effective treatment candidates and the varied sensitivity of different tumor types to ferroptosis (Chen et al., 2021). DUBs and E3 ligases are essential in controlling cells’ susceptibility to ferroptosis. They have potential as both prognostic indicators and ubiquitination pathway targets for novel small compounds, which could significantly speed up the clinical implementation of ferroptosis-based therapy. Many types of anti-cancer medications affect ubiquitin enzymes. These include inhibitors, proteolysis-targeting chimaeras (PROTACs), agonists, and molecular glues. Over the past 2 decades, we have seen substantial progress in creating E3 and DUB inhibitors, with several small compounds that target E3 or DUBs being used in preclinical trials and being improved by commercial companies. To promote the ubiquitination and proteasomal degradation of the protein of interest (POI), PROTAC, a tripartite molecule made up of an E3 ligand, a POI ligand, and a linker, binds to both E3 and POI (Schauer et al., 2019; Yang et al., 2020; Ye et al., 2021). Small compounds known as “molecular glues” can re-align ubiquitin enzymes with their substrates, triggering the breakdown of the substrates (Dale et al., 2021).

However, putting ubiquitin-based pro-ferroptosis medicines into practical use will take much work due to the complexity of ubiquitination regulation. Ubiquitin enzymes have a wide range of enzymatic activity. Therefore, it is essential to consider issues like selectivity and potential side effects. Moreover, it is essential to remember that ferroptosis is not limited to cancer cells; it also happens in other cells in the tumor microenvironment, like tumor-associated macrophages (TAMs) and T cells + CD8. These immune cells’ ability to ferroptosis can occasionally promote the growth of tumors (Xu et al., 2021). To find the most effective ubiquitin-based pro-ferroptosis therapy, it may be helpful to have a better understanding of how E3s or DUBs regulate ferroptosis.

## 6 The function of deubiquitinating enzymes (DUBs) in modulating ferroptosis

DUBs, are essential controllers of protein activity. They accomplish this by selectively eliminating ubiquitin chains from particular proteins. There are seven major groups into which these enzymes can be divided. The others are cysteine-based superfamilies, including the ubiquitin-specific proteases (USPs), Ub C-terminal hydrolases (UCHs), Machado-Joseph disease domain proteases (MJDs), motif interacting with Ub containing novel DUB family (MINDYs), ovarian tumour proteases (OTUs) and the zinc-finger and UFSP domain protein (ZUFSP) (Lange et al., 2022).

These DUBs can play various biological roles, thanks to their unique structural characteristics. The processing of ubiquitin precursors and the cleavage of poly-ubiquitin chains are two examples; trimming ubiquitin chains to protect proteins from degradation and modifying ubiquitin chains to alter the signals they transmit are two more (Cheng et al., 2019).

Recent studies have shown that DUBs regulate ferroptosis’s apoptotic process by altering substrate stability and signaling. The ways that DUBs regulate ferroptosis will be discussed in this text, along with how they might be used to establish a relationship between ferroptosis and tumor suppression. We’ll also examine the possible upsides of targeting DUBs in cancer therapies (Table 1). Shows the deubiquitinating enzymes’ regulation of ferroptosis-associated proteins.

**TABLE 1:** Deubiquitinating enzymes’ regulation of ferroptosis-associated proteins.

Deubiquitinating enzyme type	Deubiquitinating enzyme	Impact on ferroptosis	Target	Reference
USP	USP7	Promote	p53, TFRC	Tang et al. (2021a)
USP	USP7	Inhibit	hnRNPA1	Zhang et al. (2020)
USP	USP7	Promote	SLC7A11	Wang et al. (2019)
USP	USP11	Promote	Beclin1	Rong et al. (2022)
USP	USP14	Promote	IL-6, NRF2	Zhu et al. (2022)
USP	USP11	Inhibit	NRF2	Meng et al. (2021)
USP	USP14	Inhibit	Beclin1	Tsai et al. (2020)
USP	USP35	Inhibit	Ferroportin	Tang et al. (2021b)
OTU	OTUD1	Promote	IRP2, TFRC	Song et al. (2021)
USP	USP14	Promote	NCOA4	Li et al. (2021)
OTU	OTUB1	Inhibit	SLC7A11	Zhao et al. (2021)
USP	USP22	Inhibit	SLC7A11	Ma et al. (2020)
UCH	BAP1	Promote	SLC7A11	Zhang et al. (2019b)
UCH	BAP1	Promote	IP3R	Ye et al. (2022)
OTU	A20	Promote	ACSL4, SLC7A11	Gao et al. (2022a)

## 7 Additional interactions of the UPS in ferroptosis

Recent research has shown that ubiquitin-specific peptidase 11 (USP11) regulates autophagy-dependent ferroptosis in response to spinal cord ischemia-reperfusion injury by deubiquitinating Beclin 1. This connection enhances ferritin breakdown during autophagy and activates autophagy resulting in further ferroptosis (Rong et al., 2022). The process of a cell’s ferroptosis is also significantly influenced by a low-oxygen environment. Specific transcription factors susceptible to cellular hypoxia are known as hypoxia-inducible factors (HIFs), which include three subunits (HIF1A/HIF1, EPAS1/HIF2, HIF3A/HIF3, and one ARNT/HIF1 subunit). According to the findings, HIFs may play a dual role in controlling ferroptosis in various tumor types. First, HIF1A promotes the absorption as well as storage of fatty acids in human fibrosarcoma cells by inducing the development of two fatty acid-binding proteins by transcription, consequently suppressing ferroptosis triggered by RSL3 and ferroptosis activator FIN56. In contrast, EPAS1 selectively enriched polyunsaturated lipids by transactivating genes encoding hypoxia-induced lipid droplets in renal cancer cells *in vitro*, thereby promoting ferroptosis induced by ferroptosis activators RSL3, ML162, or ML210 (Miess et al., 2018; Zou et al., 2019). The VHL, a particular E3 enzyme of HIFs, acts as a direct indicator of HIF stability, which makes it an indirect regulator of ferroptosis.

**FIGURE 3:**
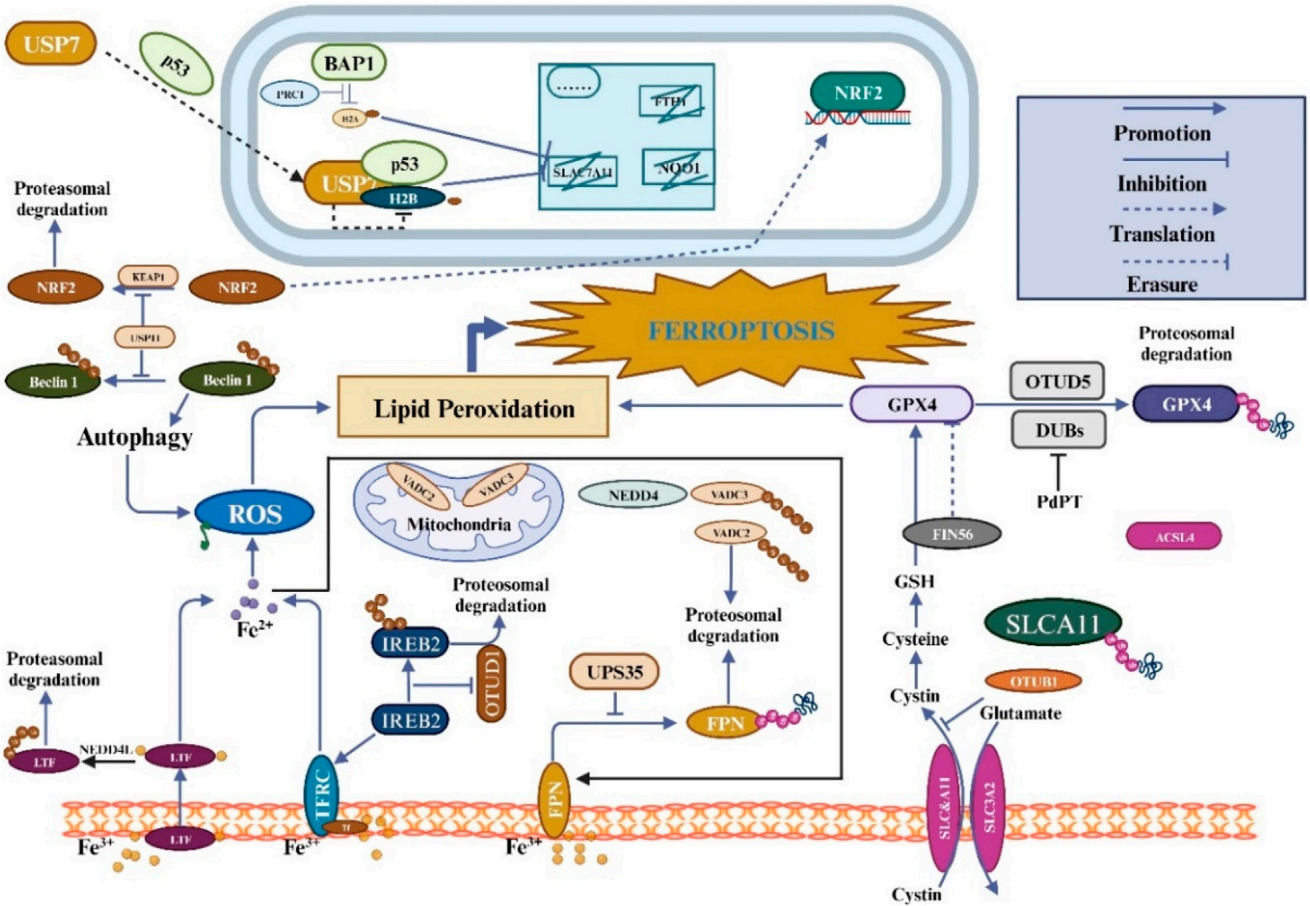
The regulation of ferroptosis involves ubiquitination and is closely linked to amino acid, iron, and lipid metabolism. Ferroptosis is induced by intracellular labile iron, which triggers lipid peroxidation. Together with GPX4 activity, cystine uptake via the System XC-transporter aids in the inhibition of ferroptosis and reduction of harmful lipid peroxides. Additionally, FSP1 and DHODH can independently inhibit ferroptosis without the involvement of GPX4. Ferroptosis regulation involves the control of these pathways via ubiquitination.

Ferroptosis depends on the stability of the functioning of mitochondria and activity because mitochondria are the primary source of RO synthesis. The volt-dependent anion channel (VDAC), a member of the multifunctional channel protein family, is essential for facilitating the passage of ions and metabolites through the outer membrane of mitochondria in eukaryotic cells (Shoshan-Barmatz et al., 2010). Studies have shown that Erastin can bind directly to VDAC2 and induce ferroptosis. When Erastin induced melanoma cells, VDAC2 and VDAC3 formed a negative feedback regulatory mechanism through proteasome-dependent degradation regulated by E3 NEDD4 (Yang et al., 2020). But more research will be needed to determine whether particular mitochondrial E3 ligases are responsible for the destruction of VDAC during ferroptosis. Recent research has shown that tumor-associated fibroblasts (CAFs) contain higher levels of Ubiquitin-specific protease-7 (USP7), which promotes the deubiquitination of miRNA sort-related protein hnRNPA1. Encourage the release of CAFs exosome miR-522, and this miR-522-secreted exosome further prevents gastric cancer cells from ferroptosizing by focusing on and suppressing the expression of ALOX15 in these cells (Zhang et al., 2020). As the understanding of ferroptosis and its mechanisms continues to improve, there is increasing evidence that members of the ubiquitin-proteasome system regulate ferroptosis and play an essential role (Figure 3). From the above, it can be found that the ubiquitin-proteasome system has a dual function in regulating ferroptosis (Table 2).

**TABLE 2:** Ferroptosis control is accomplished by the ubiquitin-proteasome system in two ways.

Name	Molecular mechanism	Promote or inhibit ferroptosis	References
OTUD5	Remove the K63 ubiquitin chain of GPX4	+	Dong et al. (2022)
OTUB1	Deubiquitination and stabilization of SLC7A11	−	Liu et al. (2019)
KEAP1	The E3 ligase adaptor of NRF2	−	Sun et al. (2016b)
USP7	p53 promotes the nuclear translocation of USP7, which Deubiq uit and stabilize of H2B	+	Wang et al. (2019)
	deubiquitination and stabilization of hnRNPA	−	Zhang et al. (2020)
BAP1	Deubiquitination and stabilization of H2A	+	Wang et al. (2004)
PRC1	Ubiquitination of H2A	−	Zhang et al. (2019a)
UbcH5c		−	Song et al. (2021)
OTUD1	Deubiquitination and stabilization of IREB2	+	Wang et al. (2020)
NEDD4L	The E3 ligase adaptor of LTF, ubiquitination of LTF	−	
USP35	Deubiquitination and stabilization of FPN	+	Tang et al. (2021b)
USP11	Deubiquitination and stabilization of NRF2	−	Meng et al. (2021)
	Deubiquitination and stabilization of Beclin 1	+	Rong et al. (2022)
VHL	The E3 ligase adaptor of HIFs	−	Zou et al. (2019)
NEDD4	The E3 ligase adaptor of VDAC2 and VDAC3	−	Yang et al. (2020b)

Note: +: promotion; −: inhibition.

## 8 Conclusion and future prospects

A unique form of cell death called ferroptosis is brought on by iron-mediated lipid peroxidation. This recently found method offers hope for eliminating cancer cells but raises an intriguing problem in developing the disease. The need for a thorough understanding of the regulatory components driving ferroptosis is further highlighted because it operates through a dualistic process, impacting distinct cells in the tumor microenvironment (TME) in various ways.

In this scenario, the post-transcriptional modification mechanism known as ubiquitination is crucial. Through substrate ubiquitination and deubiquitination regulation, this route exerts influence over a wide range of cellular processes. Cancer development and therapy both involve ubiquitin enzymes and their regulation. These enzymes interact with proteins involved in ferroptosis, which mediates cells’ sensitivity to ferroptosis and elucidates their relationship. Recent studies have started to outline how E3 ligases and DUBs collectively affect ferroptosis. However, there is still a significant gap in our knowledge of the precise mechanisms involved, such as the specifics of polyubiquitination, the specifics of ubiquitination sites, or additional procedures that are independent of ligase activity, due to the complex nature of the regulation of ubiquitin enzymes.

We currently have a limited understanding of how developing ferroptosis-regulating proteins like FSP1, DHODH, and GCH1 are regulated by E3s and DUBs. Drug development efforts to suppress ferroptosis with an E3 or DUB inhibitor will advance more quickly if these open questions are resolved. E3s and DUBs, among other ubiquitin system enzymes, are critical regulators of the ferroptosis process. Therefore, deepening our comprehension of this complex regulatory network may reveal novel cancer therapy strategies, adding a potent weapon to our therapeutic toolbox. As we get a deeper understanding of these mechanisms and their interdependencies, we may be able to shed light on hitherto uncharted avenues leading to more effective cancer therapy.
